# Supplementary material for: Determinants of stunting among adolescent girls in schools of Digo Tsion Town, Northwest Ethiopia: Unmatched case control study
Source: PLoS One. 2024 Oct 16;19(10):e0309479. doi: 10.1371/journal.pone.0309479 (PMC11482678; doi:10.1371/journal.pone.0309479)
Supplement: Attachment — Submitted filename: Reviewer 3.pdf [file pone.0309479.s005.pdf]

(Reviewer 3)

Reviewer Recommendation Term:

- ✓ Major Revision

Comments to the Author

1. If the authors have adequately addressed your comments raised in a previous round of review and you feel that this manuscript is now acceptable for publication, you may indicate that here to bypass the “Comments to the Author” section, enter your conflict of interest statement in the “Confidential to Editor” section, and submit your "Accept" recommendation.

2. Is the manuscript technically sound, and do the data support the conclusions?

The manuscript must describe a technically sound piece of scientific research with data that supports the conclusions. Experiments must have been conducted rigorously, with appropriate controls, replication, and sample sizes. The conclusions must be drawn appropriately based on the data presented.

- ✓ Partly

3. Has the statistical analysis been performed appropriately and rigorously?

- ✓ No

4. Have the authors made all data underlying the findings in their manuscript fully available?

The PLOS Data policy requires authors to make all data underlying the findings described in their manuscript fully available without restriction, with rare exception (please refer to the Data Availability Statement in the manuscript PDF file). The data should be provided as part of the manuscript or its supporting information, or deposited to a public repository. For example, in addition to summary statistics, the data points behind means, medians and variance measures should be available. If there are restrictions on publicly sharing data—e.g. participant privacy or use of data from a third party—those must be specified.

- ✓ Yes

5. Is the manuscript presented in an intelligible fashion and written in standard English?

PLOS ONE does not copyedit accepted manuscripts, so the language in submitted articles must be clear, correct, and unambiguous. Any typographical or grammatical errors should be corrected at revision, so please note any specific errors here.

- ✓ No

6. Review Comments to the Author

General comments:

- ✓ What special or unique determinants of stunting did you found as compared to different scientific evidences?
- ✓ Your recommendations were not supported by the findings of this study.

Abstract:

- ✓ Show the gaps related to your research problem
- ✓ lines 34-35: The word "face to-face interview" is not clear. did you used qualitative data collection tool? better to rephrase it.
- ✓ the conclusion part of your abstract is copy paste of your result. Better to narrate your result in different ways.

Introduction:

- ✓ You have to clearly indicated any global or national program initiatives to prevent or minimize stunting in adolescents, any success or failure and the contribution of the current study in relation to those program interventions and strategies.

Methods and Materials:

- ✓ lines 126-127: the study unit was adolescent girl not girls whether she was grouped under control or case
- ✓ lines 128-132: revise your inclusion criteria. the one you stated was already included by default.
- ✓ Lines 134-135: Also revise your exclusion criteria. why you exclude girls "spinal curvature?, who used wheelchair?" There is alternative means of height measurement for them. Did you included 1st and 2nd trimester pregnant?? why you excluded third trimester?
- ✓ Lines 197-199: Make "Wealth index" measurable. the one you stated as the operational definition of wealth index is not appropriate. After you derived the scores, How did you measured it?
- ✓ Data collection tool and procedure is too shallow. Explain the data collection tool and detail procedure for each of your independent variables like; anemia, dietary diversity, food security, presence or absence of malaria, chronic illness, and others.
- ✓ How did you directly compute VIF in logistic regression model??
- ✓ You have to describe in detail the procedure while you run Principal component analysis (PCA) like: what assumptions did you checked? what rotation did you used? How many components were extracted? what was the cut value for eigenvalues? How did you interprated the results? Finally how did you measured wealth index based on the extracted components??

Results:

- ✓ Table 3: the dietary diversity was not presented as stated on the operational definition.
- ✓ There was no any findings related to the wealth status of the study respondents. why??
- ✓ Paragraph on lines 329-334 was not needed since it was stated on the next paragraph. You have to begin the next paragraph which was on line 335, as: "The result of the multivariable analysis indicated that, Adolescent girls who ate a low-diversified diet were

nearly two (1.99) times more likely to be stunted compared with their counterparts who ate adequately diversified meals (AOR = 1.99, CI [1.06, 3.73]). --------------------------------".

- under the limitations of your study statement on lines 419-421 needs revision. "there is no anthropometric measurement scale for such a group of individuals." Are you sure??????? Think over it.
- Under recommendation, why you didn't consider institutional factors?? If you are stated as there is no any anthropometric measurement scale for individuals with spinal curvature, how did you recommended future researcher to undertake studies that are able to include those individuals with spinal curvature???
