# Supplementary material for: Determinants of stunting among adolescent girls in schools of Digo Tsion Town, Northwest Ethiopia: Unmatched case control study
Source: PLoS One. 2024 Oct 16;19(10):e0309479. doi: 10.1371/journal.pone.0309479 (PMC11482678; doi:10.1371/journal.pone.0309479)
Supplement: Attachment — Submitted filename: Response to reviewers.docx [file pone.0309479.s003.docx]

**Response for reviewers’ question**

We would like to thank both reviewers for their constructive comments and suggestions with basic questions. Next, we will respond accordingly to the reviewer’s questions. Thank you so much!

**For reviewer 1**

1. Why do not the study exclude participants suffering from other impairments that render their communication ability?

**Answer:** We did not get study participants with other impairments,

2. What type of interventions was done to protect beneficence of pregnant participants and stunted students; hence they are early to cope up with their pregnancy and to reduce the impact of the stunting, respectively?

**Answer:** There was no specific intervention given for those participants but health education on how to take adequate nutrient before, during and after pregnancy in order to prevent stunted infancy was given for the adolescent girls.

3. In order to calculate sample size, the study uses some of recently reported determining factors. I think consistency is the main additional factor to select factors to be used for sample size calculation, I doubt on using appropriate sample size calculation method.

**Answer:** Yes, agree with your concern, consistency is necessary, and we have considered in each article for its consistency, then we select only the optimal sample size from each article.

4. Why did not the study include variables that may have potential effect on the outcome variable? e.g., Grade level of the student

**Answer:** we missed these variables during tool designing by considering it will cover by age but latter we wrote it as limitation and we recommend for the next researcher.

5. There is nothing stated that for whom the interview was upheld. if the data was collected by interviewing the students, the relevance of the result will get under question mark, since the students at lower class level and the stunted respondents are not mentally competent enough to respond to the questionnaire well.

**Answer:** The interview was from both parents and the adolescent.

6. Since the problem has been studied repeatedly especially in Northern part of Ethiopia (Northwest, North, and Northeast) at where the study area of this study found, I think the result is not novel to the reader.

**Answer:** this focused on the adolescents and it is case control in study design study as well as it adds variables like coffee/tea consumption but previous studies were cross sectional in design and mainly focused on under five.

7. the relevance of the study was also compromised by involving too young age and stunted study subjects that are not mentally mature enough to respond to the interview.
   **Answer:** the interview focused both on the parents and adolescents. The adolescent is the second opportunity to prevent stunting in the population.
8. please include the specific area at where the interview was proceeded, the stunted students identified, and its status regarding privacy to give them emotional protection.

**Answer:** the interview was the same with controls since at first, they did not know in which group are them but they had code and the data collectors used that code.

**For reviewer 2**

1. Is stadiometer used for anthropometric analysis?

**Answer:** No, we have used for measuring height

2. How did you identify the cases and controls before collecting the data?

**Answer:** screening was done by measuring height using stadiometer and analysis by World Health Organization 2007software before the actual data collection period and code was given adolescents.

3. What is the difference between study population and source population for this study?

**Answer:** The study population are the students in 2022 academic year but the source populations are any school adolescent girls in the population.

4. Is it ethical to exclude those girls with spinal curvature, third trimester pregnant, who could not stand properly and walked with a wheelchair were excluded for both cases and controls? How many of these excluded from the study?

**Answer:** since we have used height measurement criteria and spinal curvature will affect the result of our measurement, so we have excluded them. And also, third trimester pregnancy also the same and the reference and analysis is different from those non pregnant adolescents. Five adolescents were excluded.

5. Sampling technique and procedures, how did you identify the cases and controls initially?

**Answer:** sampling frame was prepared during screening and finally computer-generated method was employed.

6. Stunting is a chronic condition but some of your independent variables are a short time event like drinking coffee/tea, visit by HEW’s, menstrual status. How these short time event predict the chronic condition?? How did you measure menstrual status? What did you measure?

**Answer:** yes, stunting is chronic but can be affected by different factors and drinking coffee/tea, visit by HEW’s, menstrual status was among the suspected factors due to this we wanted to assess it but it may not have direct effect on the case. Menstrual status was assessed by interviewing the adolescent girls during data collection.

**Thank you again!**
