# Supplementary material for: Determinants of stunting among adolescent girls in schools of Digo Tsion Town, Northwest Ethiopia: Unmatched case control study
Source: PLoS One. 2024 Oct 16;19(10):e0309479. doi: 10.1371/journal.pone.0309479 (PMC11482678; doi:10.1371/journal.pone.0309479)
Supplement: Attachment — Submitted filename: Response for Reviwers.docx [file pone.0309479.s007.docx]

**Response for reviewers’ question**

We would like to thank both reviewers for their constructive comments and suggestions with basic questions. Next, we will respond accordingly to the reviewer’s questions. Thank you so much!

**For Reviewer #1:**
were the newly added variables that much helpful and impactful?

**Answer: yes, but it needs follow up for the future clarification**

**For Reviewer #3:**
1. What special or unique determinants of stunting did you found as compared to different scientific evidences?

**Answer: chronic illness and drinking coffee or tea immediately while eating meals.**

2. Your recommendations were not supported by the findings of this study.
Abstract:

1. Show the gaps related to your research problem

2. lines 34-35: The word "face to-face interview" is not clear. did you used qualitative data collection tool? better to rephrase it.

3. the conclusion part of your abstract is copy paste of your result. Better to narrate your result in different ways.

Introduction:
1. You have to clearly indicated any global or national program initiatives to prevent or minimize stunting in adolescents, any success or failure and the contribution of the current study in relation to those program interventions and strategies.

**Answer: thank you for this suggestion. And we had tried to indicate in the manuscript.**

Methods and Materials:

1. lines 126-127: the study unit was adolescent girl not girls whether she was grouped under control or case

**Answer: I have revised it**

2. lines 128-132: revise your inclusion criteria. the one you stated was already included by default.

**Answer: we have revised it**

3. Lines 134-135: Also revise your exclusion criteria. why you exclude girls "spinal curvature? who used wheelchair?" There is alternative means of height measurement for them. Did you included 1st and 2nd trimester pregnant?? why you excluded third trimester?

**Answer: Those individuals who had accidental injury to their lower back had walk with wheelchair. We had excluded third trimester pregnant since we may not sure whether the adolescent is pregnant or not until last second trimester and in third trimester the adolescent’s height will be affected. In addition, this is not comparable with non-pregnant adolescents**

4. Lines 197-199: Make "Wealth index" measurable. the one you stated as the operational definition of wealth index is not appropriate. After you derived the scores, how did you measure it?

**Answer: We have already corrected on the manuscript.**

5. Data collection tool and procedure is too shallow. Explain the data collection tool and detail procedure for each of your independent variables like; anemia, dietary diversity, food security, presence or absence of malaria, chronic illness, and others.

**Answer: Yes, we agree with your concern and we have corrected the comments on the manuscript.**

6. How did you directly compute VIF in logistic regression model??

**Answer:** **We have done VIF to the correlation of bivariable outcomes with the independent factors. This can also be done through linear assumptions**.

7. You have to describe in detail the procedure while you run Principal component analysis (PCA) like: what assumptions did you checked? what rotation did you used? How many components were extracted? what was the cut value for eigenvalues? How did you inter-prated the results? Finally, how did you measured wealth index based on the extracted components??

**Answer: Thank you for your suggestion, and we have already narrated on the manuscript.**
**Results:**
1. Table 3: the dietary diversity was not presented as stated on the operational definition.

**Answer: We have already corrected**
2. There was no any findings related to the wealth status of the study respondents. why??

**Answer: There a finding but it is not associated with stunting. And we have narrated in the result part next to sociodemographic characteristics.**

3. Paragraph on lines 329-334 was not needed since it was stated on the next paragraph. You have to begin the next paragraph which was on line 335, as: "The result of the multivariable analysis indicated that, Adolescent girls who ate a low-diversified diet were nearly two (1.99) times more likely to be stunted compared with their counterparts who ate adequately diversified meals (AOR = 1.99, CI [1.06, 3.73]). --------------------------------".

**Answer: We had incorporated your suggestions**

4. under the limitations of your study statement on lines 419-421 needs revision. "There is no anthropometric measurement scale for such a group of individuals." Are you sure??????? Think over it.

**Answer: We have accepted your suggestion and We had corrected on the main manuscript. There are methods of measurement for such individuals while for the time being we had used standing stadiometers.**

5. Under recommendation, why you didn't consider institutional factors?? If you are stated as there is no any anthropometric measurement scale for individuals with spinal curvature, how did you recommended future researcher to undertake studies that are able to include those individuals with spinal curvature???

**Answer: first all we had strongly accept this comments and questions. And there are methods of measurement for such individuals while for the time being we had used only standing stadiometers due to this we had exclude at that time.**

**Thank you again!**
