# Supplementary material for: Determinants of stunting among adolescent girls in schools of Digo Tsion Town, Northwest Ethiopia: Unmatched case control study
Source: PLoS One. 2024 Oct 16;19(10):e0309479. doi: 10.1371/journal.pone.0309479 (PMC11482678; doi:10.1371/journal.pone.0309479)
Supplement: Attachment — Submitted filename: Response for reviewers.docx [file pone.0309479.s008.docx]

**Response for reviewers’ question**

We would like to thank both reviewers for their constructive comments and suggestions with basic questions. Next, we will respond accordingly to the reviewer’s questions. Thank you so much!

**For Reviewer #3:**
1. Interview is the data collection tool used for qualitative study approach. So how did you use it for your data collection?

**Answer: Interview as data collection method for both the qualitative and quantitative study approach but it differs at the time of analysis. Therefore, we can use for study.**

2. Why you exclude girls "spinal curvature? Who used wheelchair?" There is alternative means of height measurement for them. Did you include 1st and 2nd trimester pregnant?? Why you excluded third trimester? Still the response given for this issue is not scientific.

**Answer: thank you for these questions and suggestion. You are perfectly correct there is alternative methods to measure the height of those who have lower back problem. But during our screening period we had only standing stadiometers to measure the height of the girls due to this reason we forced to exclude those who have spinal curvature and who walk with wheelchair. In addition, we stated in our manuscript as a limitation this study finding. We exclude third trimester pregnant because of the increase the abdominal girth the height is affected and the result will false positive so due this better to study them separably.**

3. Make "Wealth index" measurable. The one you stated as the operational definition of wealth index is not appropriate and not measurable. After you derived the scores, how did you measure it?

**Answer: we have tried to correct in our manuscript but this depends on the wealth index tools which contains 16 questions. And this part was analysis by factor analysis (PCA).**

4. You have to describe in detail the procedure while you run Principal Component analysis (PCA) like: what assumptions did you checked? What type of rotation did you used? How many components were extracted? What was the cut value for eigenvalues? How did you interpret the results?

**Answer: thank you again for this suggestion, we have tried to describe on the manuscript.**

**For Reviewer #5:**

1. Is it case-control study or a cross-sectional study?

**Answer: It is unmatched case study**
2. More detailing will be required in the methodology portion.

**Answer: Thank you for this suggestion, we have tried to add more detain on all the reviewer concern.**
3. Why stunting is important index for assessing undernutrition and public health problems among adolescent children instead of BMI for age?

**Answer: it is possible to use both to assess the nutritional status. However, we select stunting because to begin with adolescent age group is the second opportunity to minimize the risk of malnutrition. In addition, stunting shows chronic conditions than BMI since BMI shows mainly current nutritional status than the chronic condition.**
4. Have the authors gone through the article - Hermanussen, M., & Scheffler, C. (2024). Stop stunting—A misguided campaign by well-meaning nutritionists. American Journal of Human Biology, e24068. <https://doi.org/> 10.1002/ajhb.24068

**Answer: thank you again for this information but we have seen previously even if the article is published recently and our study was conducted before this publication.**

**Thank again**

**With regards**
